# Comparative efficacy and safety of seven Chinese patent medicines combined with SSRIs for depressive disorder: a systematic review and network meta-analysis

**DOI:** 10.3389/fpsyt.2026.1872413

**Published:** 2026-08-14

**Authors:** Hongye Geng, Lixuan Li, Chengyuan Lu, Shuling Wang

**Affiliations:** School of Business Administration, Shenyang Pharmaceutical University, Shenyang, China

**Keywords:** adjunctive therapy, Chinese patent medicines, depressive disorder, network meta- analysis, selective serotonin reuptake inhibitors, systematic review

## Abstract

**Background:**

Depressive disorder is a leading cause of disability worldwide, and selective serotonin reuptake inhibitors (SSRIs) remain first-line pharmacotherapy. However, the efficacy and safety of adding Chinese patent medicines (CPMs) as adjunctive therapy to SSRIs have not been systematically evaluated in a network meta-analysis.

**Objective:**

To compare and rank seven CPMs combined with SSRIs for depressive disorder in terms of HAMD improvement, clinical response rate, and adverse drug reactions (ADRs) rate.

**Methods:**

We searched MEDLINE, EMBASE, PsycINFO, CENTRAL, CNKI, WanFang, and VIP from inception to April 1, 2026. Trials comparing any CPM + SSRI versus the same SSRI alone in adults with depressive disorder were included. Two authors independently extracted data and assessed risk of bias (RoB 2.0). Pairwise meta-analysis and network meta-analysis (using both netmeta and getmc packages) were performed; cumulative probability (SUCRA) rankings were derived from Bayesian analysis with getmc. Confidence in evidence was evaluated with CINeMA.

**Results:**

Sixty-five trials involving 5,975 patients were included. Compared with SSRIs alone, all CPM + SSRI combinations significantly improved HAMD score (MD range –2.60 to –6.20) and clinical response rate (OR range 1.8–5.0). For HAMD, JYW+SSRIs showed the highest SUCRA ranking, followed by BJTGT+SSRIs and SGJY+SSRIs, though these rankings are probabilistic. For response rate, BJTGT+SSRIs showed the highest SUCRA ranking, but statistically unconfirmed. For safety, TM+SSRIs showed the highest SUCRA ranking, though no ADR comparison reached significance; YXQN+SSRIs ranked second in SUCRA analysis, based on one small trial. Egger’s test indicated no publication bias for HAMD (p = 0.5753) but potential bias for clinical response rate (p = 0.0002). Sensitivity analysis excluding small studies confirmed the robustness of HAMD findings.

**Conclusion:**

Adding CPMs to SSRIs may enhance efficacy and reduce ADRs. Although the rankings suggest promising efficacy, they are probabilistic in nature, and the effect estimates and their confidence intervals should be given greater weight in interpretation. No ADR comparisons reached statistical significance, and evidence supporting the safety and efficacy of some CPMs remains limited. Findings should be interpreted with caution, and future studies should further evaluate the safety and efficacy of combined therapy.

**Systematic review registration:**

https://www.crd.york.ac.uk/prospero/, identifier CRD420261299414.

## 1 Introduction

Depressive disorder is a mounting global public health issue. It is characterized by persistent low mood, anhedonia, cognitive impairment, sleep disturbance, fatigue, and somatic discomfort (1, 2). It constitutes a leading cause of disability, impaired quality of life, and suicide worldwide, imposing a heavy disease burden on individuals and society (3). According to the International Classification of Diseases (11th rev.; ICD-11) (4), the diagnosis of depressive disorder requires the presence of specific symptom criteria over a defined period. The pathophysiology involves multiple pathways, including neurotransmitter imbalance, neuroinflammation, impaired neuroplasticity, and dysregulation of the hypothalamic–pituitary–adrenal axis (5).

First-line medications include SSRIs, SNRIs, and NARIs, which exert antidepressant effects mainly by blocking neurotransmitter reuptake (6). Among these, SSRIs have the greatest clinical value due to their tolerability and safety profile (7). However, conventional antidepressants have critical limitations, including inadequate response in some patients and severe adverse effects in others (8). Thus, safer, faster-onset, and more effective adjuvant therapies are urgently needed (9).

Chinese patent medicines (CPMs) have been increasingly used as complementary therapy for depression, with potential advantages of multi-target regulation, fewer side effects, and synergistic effects with SSRIs (10, 11). A growing number of trials have evaluated the efficacy and safety of CPMs combined with SSRIs, suggesting that combined therapy may improve response rates, accelerate symptom relief, and reduce adverse drug reactions (12). Nevertheless, clinical evidence regarding the comparative effectiveness of different CPM–SSRI combinations remains inconsistent, and head-to-head trials are scarce. Most previous traditional meta-analyses focused on pairwise comparisons of non-pharmacological therapies, or Chinese, or Western medicine alone, whereas the combination of Chinese and Western medicine remains understudied (13, 14). In addition, earlier reviews are outdated and fail to incorporate newly published high-quality trials, leading to low certainty of evidence.

Therefore, we conducted a systematic review and network meta-analysis (NMA) to evaluate and update the efficacy, especially the early-phase effect, and safety of CPMs combined with SSRIs for depressive disorder by including the latest trial data. This study aims to compare and rank the comparative effectiveness of different adjuvant CPM regimens and provide reliable evidence for clinical decision-making.

## 2 Article types

This network meta-analysis was conducted in accordance with the PRISMA 2020 guidelines (15) and has been prospectively registered on PROSPERO (CRD420261299414).

### 2.1 Criteria for considering studies for this review

#### 2.1.1 Types of studies

We included parallel-group trials regardless of publication status or language. Cluster-trials, controlled before-and-after studies, case reports, cohort studies, and other non-randomized designs were excluded.

#### 2.1.2 Types of participants

We included adult patients (≥18 years) diagnosed with depressive disorder using ICD-10, DSM-5, CCMD-3, or other validated standardized tools. No restrictions were applied regarding sex, disease duration, or TCM syndrome types. Trials on patients with comorbidities were also included as long as depression was the main focus.

#### 2.1.3 Types of interventions

Experimental interventions: CPMs combined with SSRIs. Eligible CPMs included Shugan Jieyu Capsule, Bajitian Oligosaccharide Capsule, Wuling Capsule, Jieyu Wan, Xiaoyao Wan, Tianmeng Oral Liquid, Yangxue Qingnao Granule, and other officially marketed patent medicines.

Comparator interventions: The same SSRI alone or SSRI plus placebo. Studies using SNRIs, NaSSA, or other non-SSRI antidepressants were excluded.

#### 2.1.4 Types of outcome measures

- Primary efficacy outcome: Depression severity scores (continuous), prioritized as HAMD (17- or 24-item) > MADRS > SDS > other validated scales.
- Secondary efficacy outcome: Clinical response rate (dichotomous).
- Safety outcome: Total number of adverse drug reactions (ADRs)
- Outcomes were extracted at the end of treatment.

### 2.2 Search methods for identification of studies

MEDLINE, EMBASE, PsycINFO, CENTRAL (via OVID and Cochrane Library), CNKI, WanFang Data, and VIP were searched from inception to April 1, 2026, without language restrictions. Reference lists of retrieved articles were also screened. The full search strategy is provided in Supplementary material.

### 2.3 Data collection and analysis

Two independent reviewers (GH and LL) screened titles/abstracts and full texts based on the inclusion criteria. Disagreements were resolved by discussion or referral to a third reviewer (LC). Data extraction included trial characteristics, participant demographics, intervention details, and outcome data. If published data were insufficient, corresponding authors were contacted.

### 2.4 Risk of bias assessment

Using the RoB 2.0 tool (16), two authors (GH and LL) independently assessed each included trial across five domains: randomization process, deviations from intended interventions, missing outcome data, measurement of the outcome, and selection of the reported result. Each domain was rated as low risk, some concerns, or high risk. An overall risk of bias was also assigned.

### 2.5 Measures of treatment effect

For continuous outcomes (HAMD score changes), we used mean difference (MD) with 95% confidence interval (CI). For dichotomous outcomes of adverse drug reactions (ADRs) rate, we used odds ratio (OR) with 95% CI. A p-value < 0.05 was considered statistically significant.

### 2.6 Dealing with missing data

We contacted original authors for missing data. If unavailable, we used only the available data without imputation.

### 2.7 Assessment of heterogeneity

Heterogeneity was assessed using the I² statistic and chi-square test. The criteria were: I² < 40% (low), 30-60% (moderate), 50-90% (substantial), and 75-100% (considerable).

### 2.8 Assessment of reporting biases

Publication bias was evaluated visually using comparison-adjusted funnel plots and quantitatively using Egger’s regression test. A p-value > 0.05 for the intercept indicated no statistically significant asymmetry.

### 2.9 Statistical analysis

NMA was performed using both frequentist and Bayesian approaches. The frequentist NMA was conducted with the netmeta package (version 3.2-0) in R, and the Bayesian NMA was carried out using the getmc package under a random-effects model. The frequentist method was used for primary effect estimates, while the Bayesian approach with non-informative priors (normal prior for log-OR/MD, uniform prior for heterogeneity τ) was used to derive SUCRA rankings. Four MCMC chains were run with 20,000 iterations each, with the first 5,000 discarded as burn-in. The transitivity assumption was evaluated by comparing the distribution of effect modifiers across comparisons; distributions were broadly similar. For consistency assessment, node-splitting and design-by-treatment interaction methods were applied when closed loops existed; in the absence of closed loops, a consistency model was applied by default. Both direct and indirect evidence were synthesized to obtain relative effect estimates for all possible pairwise comparisons. Continuous outcomes (HAMD score changes) were analyzed as MDs with 95% CIs. Dichotomous outcomes (clinical response rate and ADR incidence) were analyzed as ORs with 95% CIs. Heterogeneity in the network was assessed using the I² statistic derived from the network model. League tables were generated for each outcome. Treatment rankings were derived from the Bayesian NMA as surface under the cumulative ranking curve (SUCRA) values, which reflect the probability of each intervention being among the best. Higher SUCRA values indicate better efficacy for HAMD and response rate, and lower ADR risk for safety. Cumulative probability plots were also generated to visualize ranking distributions. Publication bias and small-study effects were assessed using comparison-adjusted funnel plots and Egger’s regression test for outcomes with ten or more studies. Sensitivity analysis was conducted by excluding studies with a sample size ≤ 50 participants per arm, studies at high RoB (RoB 2.0), and studies with intervention duration < 4 weeks. The quality of evidence for primary outcomes was evaluated using the Confidence in Network Meta-Analysis (CINeMA) framework, which considers six domains: within-study bias, reporting bias, indirectness, imprecision, heterogeneity, and inconsistency. The CINeMA web application was used to generate summary ratings, categorizing the credibility of results as high, moderate, low, or very low.

## 3 Results

### 3.1 Description of studies

#### 3.1.1 Results of the search

**Figure 1:**
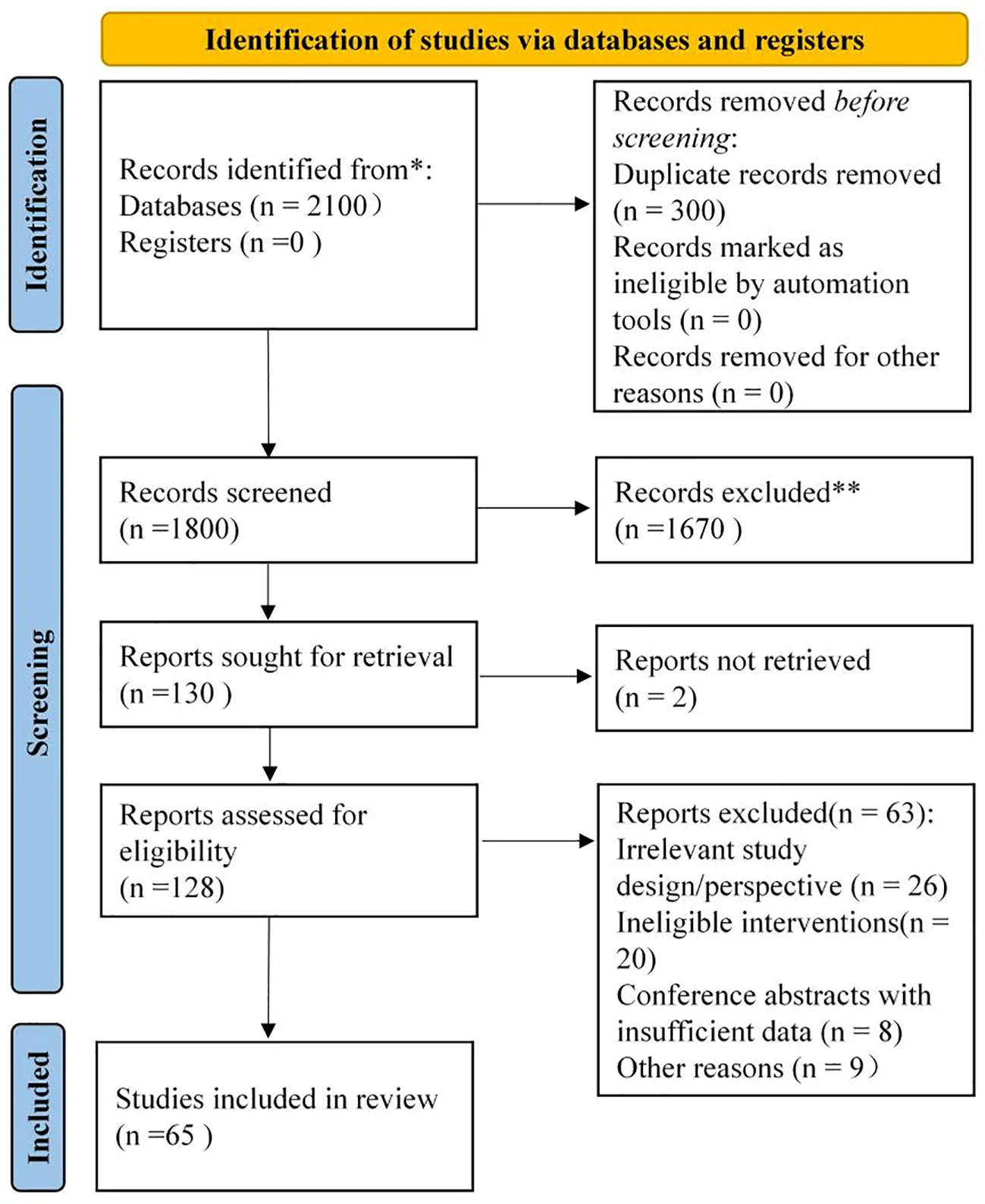
PRISMA 2020 flow diagram of the study selection process.

A total of 2,100 records were identified. After removing 300 duplicates, 1,800 records were screened. Of these, 1,670 records were excluded based on title and abstract. Full texts of 130 reports were sought, and 2 reports were unavailable. The remaining 128 full-text articles were assessed for eligibility, and 63 were excluded (irrelevant design, n = 26; ineligible interventions, n = 20; conference abstracts, n = 8; other reasons, n = 9). Consequently, 65 trials (17–81)were included in the qualitative and quantitative synthesis (**Figure 1**).

#### 3.1.2 Study characteristics

The 65 trials included 5,975 participants (range 40–300). All were two-arm, parallel-group trials conducted in China. Diagnoses included depressive disorder (51 trials), treatment-resistant depression (6), recurrent depressive disorder (1) (20), depressive episode (3) (42, 61, 72), depression with comorbid anxiety (3), and depression with insomnia (1) (31). Diagnostic criteria varied: CCMD-3 (15 trials), ICD-10 (12), DSM-IV/5 (3) (32, 35, 60), combination criteria (22), and HAMD-only (13).

Interventions: Shugan Jieyu Capsule (33 trials), Jieyu Wan (7), Bajitian Oligosaccharide Capsule (5), Wuling Capsule (6), Xiaoyao Wan (10), Tianmeng Oral Liquid (3) (36, 61, 62), and Yangxue Qingnao Granule (1) (76). SSRIs used: escitalopram (20 trials), paroxetine (16), sertraline (11), fluoxetine (10), citalopram (4), fluvoxamine (4). Treatment duration: 4 weeks (10), 6 weeks (30 trials), 8 weeks (12), 10 weeks (5), 12 weeks (5). All trials reported HAMD; 58 reported response rate; 49 reported adverse drug reactions (ADRs) (**Supplementary Table 1**).

#### 3.1.3 Risk of bias in included studies

Two independent authors assessed the RoB (**Supplementary Figures 1**, **2**). For the randomization process, 15 trials were low risk, 50 had some concerns, and none were high risk. For deviations from intended interventions, none were low risk, 65 had some concerns, and none were high risk. For missing outcome data, 64 were low risk, none had some concerns, and one was high risk. For measurement of the outcome, 62 were low risk, 3 had some concerns, and none were high risk. For selection of the reported result, 47 were low risk, 18 had some concerns, and none were high risk. Overall, one trial was low risk, 63 had some concerns, and one was high risk.

In summary, most trials had some concerns in the randomization process and selection of the reported result, while the RoB for missing outcome data and outcome measurement was low.

### 3.2 Pairwise meta-analysis

The pairwise meta-analysis for HAMD, overall response rate, and total adverse drug reactions (ADRs) rate is presented in (**Supplementary Tables 2A–C**).

All six Chinese herbal preparations combined with SSRIs significantly improved the overall response rate compared with SSRIs alone (OR = 2.49–3.60; all P < 0.05). Among them, Shugan Jieyu capsule + SSRIs (OR = 3.12 [2.64, 3.70]; p < 0.001), Wuling capsule + SSRIs (OR = 3.41 [2.49, 4.66]; p < 0.001), and Bajitian Oligosaccharide Capsule + SSRIs (OR = 3.60 [1.60, 8.07]; p = 0.012) showed the largest effect sizes.

For HAMD reduction, Shugan Jieyu capsule + SSRIs (MD = –3.54 [–4.25, –2.83]; p < 0.001), Wuling capsule + SSRIs (MD = –2.30 [–3.29, –1.31]; p = 0.003), Jieyu pill + SSRIs (MD = –4.96 [–8.51, –1.40]; p = 0.014), and Xiaoyao pill + SSRIs (MD = –2.63 [–3.62, –1.64]; p < 0.001) all significantly improved depressive symptoms, while Bajitian Oligosaccharide capsule + SSRIs (MD = –3.61 [–7.38, 0.16]; p = 0.056) and Tianmeng Oral liquid + SSRIs (MD = –1.20 [–3.84, 1.44]; p = 0.189) did not reach statistical significance.

Regarding safety, Shugan Jieyu capsule + SSRIs (OR = 0.75 [0.57, 0.98]; p = 0.034) and Xiaoyao pill + SSRIs (OR = 0.43 [0.30, 0.62]; p = 0.001) significantly reduced the total ADR rate compared with SSRIs alone, whereas other combinations showed no significant difference.

### 3.3 NMA results

#### 3.3.1 Network evidence plot

**Figure 2:**
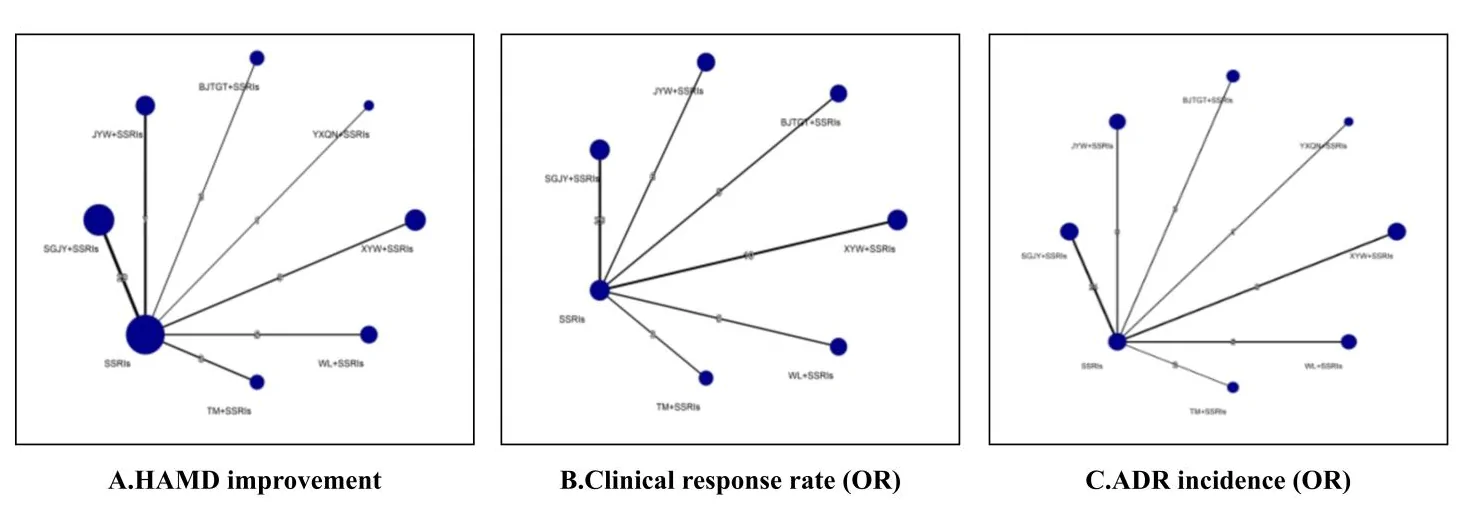
Network evidence plots for the three outcomes: **(A)** HAMD improvement (57 trials, 5,157 patients, seven CPM + SSRI regimens); **(B)** clinical response rate (58 trials, 5,201 patients, six regimens); **(C)** adverse drug reactions (ADRs) incidence (49 trials, 4,695 patients, seven regimens).

**Figure 2A** presents the network of evidence for the HAMD score. A total of 57 trials involving 5,157 patients were included for this outcome, evaluating seven treatment regimens combining Chinese patent medicines with SSRIs: SGJY+SSRIs, JYW+SSRIs, BJTGT+SSRIs, YXQN+SSRIs, XYW+SSRIs, WL+SSRIs, and TM+SSRIs. The network structure showed no closed loops between interventions; all comparisons were indirectly connected via the common comparator (SSRIs monotherapy). Therefore, a consistency model was applied for analysis, and no inconsistency test was required.

**Figure 2B** presents the network of evidence for the response rate. A total of 58 trials involving 5,201 patients were included for this outcome, evaluating six treatment regimens combining Chinese patent medicines with SSRIs: SGJY+SSRIs, JYW+SSRIs, BJTGT+SSRIs, XYW+SSRIs, WL+SSRIs, and TM+SSRIs. The network structure showed no closed loops between interventions; all comparisons were indirectly connected via the common comparator (SSRIs monotherapy). Therefore, a consistency model was applied for analysis, and no inconsistency test was required.

**Figure 2C** presents the network of evidence for the incidence of adverse drug reactions. A total of 49 trials involving 4,695 patients were included for this outcome, evaluating seven treatment regimens combining Chinese patent medicines with SSRIs: SGJY+SSRIs, JYW+SSRIs, BJTGT+SSRIs, YXQN+SSRIs, XYW+SSRIs, WL+SSRIs, and TM+SSRIs. The network structure showed no closed loops between interventions; all comparisons were indirectly connected via the common comparator (SSRIs monotherapy). Therefore, a consistency model was applied for analysis, and no inconsistency test was required.

#### 3.3.2 League table

The league tables for HAMD, response rate, and ADR incidence are presented in **Tables 1**, **2**, respectively. For HAMD, significant pairwise comparisons (95% CI excluding 0) were: BJTGT+SSRIs vs. SSRIs (MD = –3.58, 95% CI: –6.58 to –0.58), JYW+SSRIs vs. SSRIs (–4.99, –7.15 to –2.84), SGJY+SSRIs vs. SSRIs (–3.55, –4.61 to –2.49), and XYW+SSRIs vs. SSRIs (–2.65, –4.49 to –0.82).For response rate, SSRIs was significantly inferior to BJTGT+SSRIs (OR = 0.32, 95% CI: 0.20–0.53), SGJY+SSRIs (0.34, 0.27–0.43), and JYW+SSRIs (0.35, 0.22–0.55), but significantly superior to WL+SSRIs (3.18, 1.90–5.31), XYW+SSRIs (2.69, 1.80–4.01), and TM+SSRIs (2.38, 1.35–4.18).

**Table 1:** League table for HAMD improvement (MD, 95% CI).

**BJTGT+SSRIs**							
-1.42 [-5.11, 2.27]	**JYW+SSRIs**						
0.03 [-3.15, 3.21]	1.45 [-0.95, 3.85]	**SGJY+SSRIs**					
3.58 [0.58, 6.58]	4.99 [2.84, 7.15]	3.55 [2.49, 4.61]	**SSRIs**				
1.46 [-2.68, 5.60]	2.88 [-0.70, 6.45]	1.43 [-1.62, 4.47]	-2.12 [-4.97, 0.73]	**TM+SSRIs**			
1.12 [-2.83, 5.08]	2.54 [-0.82, 5.90]	1.10 [-1.69, 3.88]	-2.45 [-5.03, 0.13]	-0.33 [-4.18, 3.51]	**WL+SSRIs**		
0.92 [-2.60, 4.44]	2.34 [-0.49, 5.17]	0.89 [-1.23, 3.02]	-2.65 [-4.49, -0.82]	-0.54 [-3.93, 2.86]	-0.20 [-3.37, 2.96]	**XYW+SSRIs**	
1.68 [-4.76, 8.11]	3.09 [-2.99, 9.18]	1.65 [-4.14, 7.44]	-1.90 [-7.59, 3.79]	0.22 [-6.15, 6.58]	0.55 [-5.70, 6.80]	0.75 [-5.23, 6.73]	**YXQN+SSRIs**

Values in the upper-right triangle represent mean differences versus SSRIs monotherapy; values in the lower-left triangle represent pairwise comparisons between CPM+SSRI combinations. Negative values favor the column intervention.

Bold values indicate 95% CI does not include 0.

**Table 2:** League table for clinical response rate and ADR incidence (OR, 95% CI).

Clinical response rate							
**BJTGT+SSRIs**	0.93 [0.47, 1.82]	0.95 [0.56, 1.62]	0.32 [0.20, 0.53]	0.77 [0.36, 1.63]	1.03 [0.51, 2.09]	0.87 [0.46, 1.64]	-
0.77 [0.29, 2.05]	**JYW+SSRIs**	1.02 [0.61, 1.70]	0.35 [0.22, 0.55]	0.83 [0.40, 1.72]	1.11 [0.56, 2.21]	0.94 [0.51, 1.73]	-
0.71 [0.30, 1.67]	0.92 [0.50, 1.72]	**SGJY+SSRIs**	0.34 [0.27, 0.43]	0.81 [0.44, 1.49]	1.08 [ 0.62, 1.90]	0.92 [0.58, 1.45]	-
0.53 [0.24, 1.19]	0.69 [0.39, 1.20]	0.74 [0.56, 0.98]	**SSRIs**	2.38 [1.35, 4.18]	3.18 [1.90, 5.31]	2.69 [1.80, 4.01]	-
0.98 [0.24, 3.99]	1.27 [0.36, 4.56]	1.38 [0.42, 4.49]	1.86 [0.59, 5.84]	**TM+SSRIs**	1.34 [0.62, 2.87]	1.13 [0.57, 2.26]	-
0.90 [0.33, 2.42]	1.16 [0.52, 2.60]	1.26 [0.66, 2.39]	1.69 [0.95, 3.02]	0.91 [0.25, 3.30]	**WL+SSRIs**	0.85 [0.44, 1.62]	-
1.19 [0.48, 2.97]	1.54 [0.76, 3.13]	1.67 [1.00, 2.79]	2.25 [1.46, 3.46]	1.21 [0.36, 4.12]	1.33 [0.64, 2.74]	**XYW+SSRI**s	-
1.66 [0.35, 7.77]	2.15 [0.51, 9.00]	2.33 [0.61, 8.95]	3.13 [0.84, 11.70]	1.69 [0.29, 9.68]	1.85 [0.44, 7.81]	1.39 [0.35, 5.58]	**YXQN+SSRIs**
**ADR incidence**							

Bold values indicate 95% CI does not include 1.

For ADR incidence, none of the comparisons reached statistical significance, but TM+SSRIs showed the highest SUCRA ranking in the safety ranking analysis.

#### 3.3.3 SUCRA ranking plot (cumulative probability)

**Figure 3:**
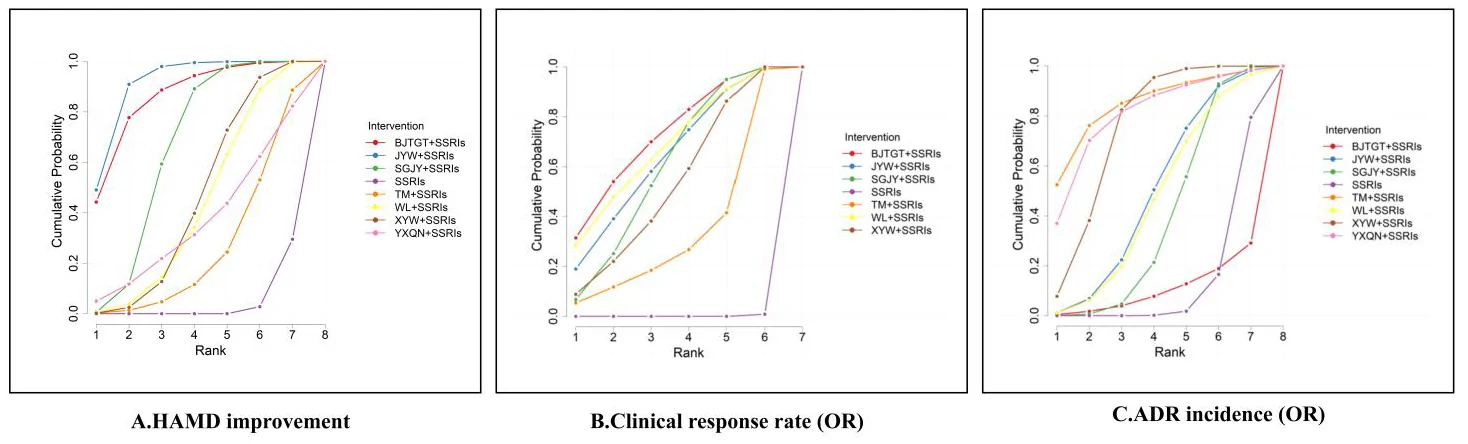
Cumulative probability (SUCRA) plots from the Bayesian network meta-analysis for **(A)** HAMD improvement, **(B)** clinical response rate, and **(C)** adverse drug reactions (ADRs) incidence.

To compare the relative effectiveness and safety of all interventions across three outcomes, we generated cumulative probability curves based on the Bayesian NMA using the getmc package (**Figures 3A–C**). The area under each curve corresponds to the SUCRA value, with higher values indicating a better ranking.

HAMD improvement (**Figure 3A**): JYW+SSRIs showed the highest SUCRA ranking, followed by BJTGT+SSRIs, SGJY+SSRIs, and XYW+SSRIs. SSRIs alone had the lowest SUCRA ranking.

Response rate (**Figure 3B**): BJTGT+SSRIs showed the highest SUCRA ranking, then WL+SSRIs and JYW+SSRIs.

ADR incidence (**Figure 3C**): Based on the posterior ranking probabilities, TM+SSRIs showed the highest SUCRA ranking for safety, followed by YXQN+SSRIs and XYW+SSRIs. However, none of the pairwise comparisons for ADR incidence reached statistical significance. The evidence for YXQN+SSRIs is limited to a single small trial, compromising the precision and generalizability of its safety estimate. TM+SSRIs was supported by a larger body of evidence, but its safety ranking is also derived from non-significant comparisons. Both safety rankings should therefore be regarded as exploratory.

These results indicate that interventions SUCRA ranking highest for efficacy are not necessarily those ranking highest for safety, highlighting the need for a benefit–risk assessment.

### 3.4 Heterogeneity

Heterogeneity was low to moderate for most comparisons (I² range 0–45%). For HAMD improvement, some comparisons showed substantial heterogeneity (I² = 92.5%), which was explored through sensitivity analysis.

### 3.5 Reporting bias

**Figure 4:**
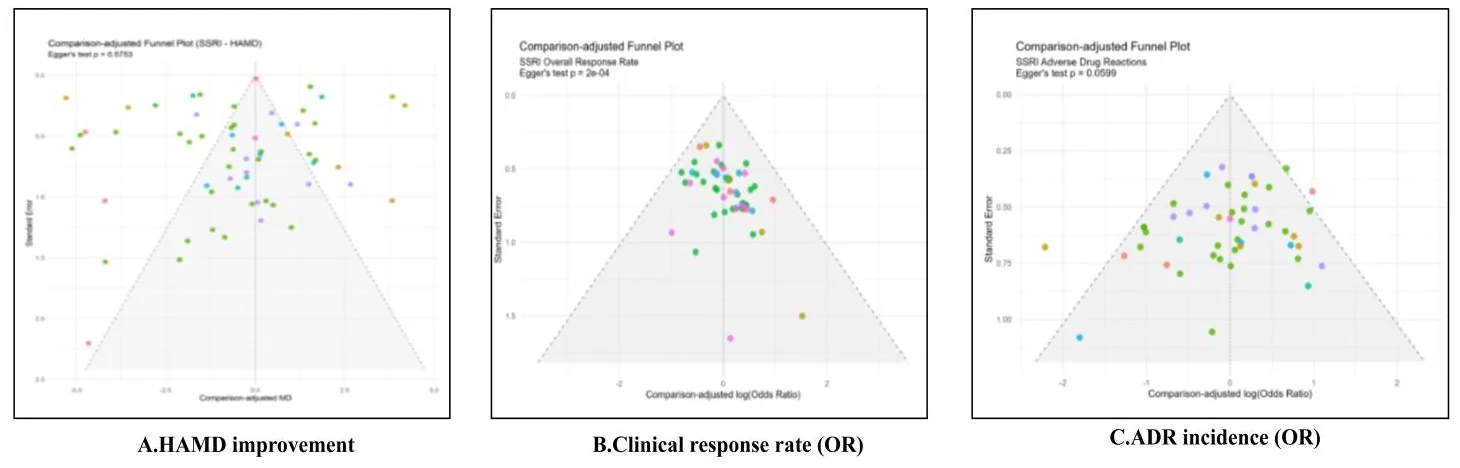
Comparison-adjusted funnel plots assessing publication bias and small-study effects for **(A)** HAMD improvement (Egger’s test p = 0.5753), **(B)** clinical response rate (p = 0.0002), and **(C)** adverse drug reactions (ADRs) incidence (p = 0.0599).

We assessed publication bias using comparison-adjusted funnel plots and Egger’s test (**Figures 4A–C**).

HAMD score improvement (**Figure 4A**): The funnel plot was roughly symmetric; Egger’s test p = 0.5753, indicating no significant publication bias.

Overall response rate (**Figure 4B**): Noticeable asymmetry; Egger’s test p = 0.0002, suggesting potential publication bias or small-study effects. Results should be interpreted cautiously.

ADR incidence (**Figure 4C**): The test was borderline significant (P = 0.0599), suggesting possible asymmetry, so modest publication bias cannot be ruled out.

### 3.6 Sensitivity analysis

To assess the robustness of the Bayesian estimates and to identify whether any single study drove the overall results for the HAMD outcome (which showed substantial heterogeneity, I² = 92.5%), we performed a leave-one-out sensitivity analysis. Specifically, we sequentially excluded each individual study and re-ran the Bayesian NMA. In all leave-one-out iterations, the direction and magnitude of the effect estimates for each intervention relative to SSRIs remained consistent with the original analysis, indicating that no single study dominated the overall findings. These results support the robustness of the HAMD findings despite the substantial heterogeneity observed.

## 4 Discussion

This systematic review and network meta-analysis provides a comprehensive comparison of seven Chinese patent medicines combined with SSRIs for depressive disorder. Our results indicate that all CPM + SSRI combinations were associated with improvements in HAMD scores and clinical response rates compared with SSRIs alone, and most showed favorable trends for reducing adverse drug reactions (ADRs) risk. The SUCRA rankings underpinning these results should be interpreted cautiously and considered alongside the effect estimates and their associated uncertainty, as SUCRA values reflect probabilities, not the magnitude of clinical benefit.

JYW+SSRIs showed the highest SUCRA ranking for HAMD improvement. Jieyu Wan, a multi-herbal formula containing active compounds such as quercetin (from *Bupleurum chinense*) and berberine (from *Coptis chinensis*), has shown antidepressant efficacy consistent with its traditional use in soothing liver qi and relieving mental distress. Mechanistically, it may act on 20 depression-related genes (e.g., GSK3B, NR3C1, SLC6A4), leading to the regulation of serotonergic/dopaminergic transmission, suppression of neuroinflammation through MAPK14/NF-κB and Ca²^+^/cAMP-PKA signaling, and modulation of HPA axis activity. Additionally, its systemic effects extend to alleviating comorbid somatic symptoms such as insomnia (82). These mechanistic hypotheses are not directly tested in the current network meta-analysis and require validation in future translational research. These multi-target mechanisms may partly account for its favorable ranking for core depressive symptoms.

BJTGT+SSRIs (Bajitian Oligosaccharide Capsule) showed a favorable SUCRA ranking for clinical response rate. This finding aligns with the traditional use of *Morinda officinalis* (Bajitian) as a kidney-tonifying and antidepressant herb. Preclinical studies suggest that oligosaccharides from *Morinda officinalis* enhance neuroplasticity via BDNF-TrkB signaling and modulate the hypothalamic–pituitary–adrenal (HPA) axis (83).These between-outcome differences suggest that the two measures capture different dimensions of treatment benefit: response rate reflects a dichotomous clinical judgment that may be sensitive to early, non-specific improvements, while HAMD offers a more comprehensive and multidimensional symptom assessment.

For ADR incidence, TM+SSRIs (Tianmeng Oral Liquid) showed the highest SUCRA ranking for safety, followed by YXQN+SSRIs (Yangxue Qingnao Granule). Previous literature has hypothesized that TM+SSRIs contain Gastrodia and Zizyphus, which have neuroprotective and anti-inflammatory properties (84). Although numerically lower incidence of gastrointestinal or neurological side effects was observed compared with other CPMs, these differences were not statistically significant. Notably, no adverse drug reaction comparison reached statistical significance in this analysis. These mechanistic hypotheses are not directly tested in the current network meta-analysis and require validation in future translational research. However, YXQN+SSRIs was evaluated in only a single trial with a small sample size. This limited evidence base compromises the precision and generalizability of its safety estimate, and further large-scale trials are needed to confirm its safety advantage.

Our findings indicate that rankings for efficacy and safety often diverged across interventions. For example, JYW+SSRIs showed a favorable SUCRA ranking for HAMD improvement but a more moderate ranking for safety, whereas TM+SSRIs showed the opposite pattern. Several other CPMs also showed inconsistent rankings across outcomes; for instance, WL+SSRIs ranked higher for response rate than for HAMD reduction. This may be because response rate is often measured at 6–8 weeks and relies on a 50% HAMD reduction threshold, which can be reached through early improvements in sleep or anxiety that are not fully captured by the total HAMD score. Similarly, XYW+SSRIs showed a higher SUCRA ranking for safety than for efficacy, possibly because its proposed mechanism (regulating the HPA axis and reducing inflammation) may produce gradual rather than rapid symptom relief. These mechanistic hypotheses are not directly tested in the present NMA and require validation in future research. These differences underscore the importance of selecting outcome measures that reflect the specific therapeutic goals for each patient. Given the exploratory nature of the ranking analysis and the low certainty of the evidence, these findings should not be interpreted as supporting the superiority of any specific intervention.

Overall, the pairwise and network meta-analysis results were directionally consistent: all CPM + SSRI combinations showed favorable trends compared with SSRIs alone for HAMD and response rate, and most for ADR. Minor numerical differences were observed for interventions with limited direct evidence (e.g., BJTGT+SSRIs and TM+SSRIs), which is expected because network meta-analysis borrows strength from indirect comparisons. For ADR, TM+SSRIs showed a non-significant favourable trend in pairwise analysis (OR = 0.55, 95% CI extremely wide) and showed the highest SUCRA ranking in the network. No substantial conflict was detected.

Our sensitivity analysis, which excluded small studies (n < 50 per arm), revealed that the ranking for response rate changed substantially: SGJY+SSRIs became the top-ranked intervention, and JYW+SSRIs lost statistical significance. This indicates that small-study effects potentially influenced the response rate results, as also suggested by the significant Egger’s test (p = 0.0002). In contrast, the HAMD outcome remained stable, supporting its robustness as the primary efficacy endpoint. Therefore, the response rate findings should be interpreted with caution, whereas the HAMD rankings are more reliable.

The significant publication bias for response rate (Egger test P = 0.0002) is a critical concern. It suggests that small or negative studies may be missing, leading to potentially overestimated odds ratios. This further supports our decision to prioritize the HAMD outcome (which showed no significant bias, p = 0.5753) as the primary efficacy endpoint.

Our findings extend previous pairwise meta-analyses that evaluated individual CPMs combined with SSRIs. For example, a meta-analysis of Shugan Jieyu Capsule (85) reported favourable effects on HAMD, consistent with our ranking. To our knowledge, few network meta-analyses have simultaneously ranked multiple CPMs for both efficacy and safety in depressive disorder; our findings provide a preliminary evidence synthesis that may help generate hypotheses for future research.

The substantial heterogeneity observed (e.g., HAMD improvement I² = 92.5%) likely stems from multiple clinical factors: (1) diagnostic criteria varied across studies (CCMD-3, ICD-10, DSM-IV/5, and HAMD-only criteria); (2) different SSRI types (escitalopram, paroxetine, sertraline, fluoxetine, citalopram, fluvoxamine) have distinct pharmacodynamic profiles; (3) treatment duration ranged from 4 to 12 weeks; (4) some trials specified TCM syndrome differentiation criteria while others did not. Due to limited reporting in primary studies, we were unable to perform subgroup analyses to identify the sources of heterogeneity.

Several limitations should be acknowledged. First, most included trials had “some concerns” in risk of bias (RoB 2.0), primarily due to inadequate reporting of randomization and blinding. This reduces the certainty of evidence. Second, heterogeneity was high to substantial for some outcomes (e.g., response rate I² = 98.2%), likely because of differences in baseline severity, treatment duration, and outcome instruments. Third, all trials were conducted in China, which may limit generalizability to other ethnic or healthcare settings. Fourth, the evidence for YXQN+SSRIs is based on a single small trial, making its safety ranking preliminary. Fifth, the ADR network was sparse, and none of the pairwise comparisons reached statistical significance, so the safety rankings are exploratory. Sixth, we could not perform subgroup analyses by depression severity, duration, or SSRI type due to insufficient data. Seventh, sensitivity analysis could only be performed for response rate because of the limited number of trials for other outcomes. Finally, the certainty of evidence assessed by CINeMA was low to moderate for most comparisons.

The overall quality of included trials was suboptimal. According to the RoB 2 assessment, only one study was rated as low risk of bias, while 63 trials had “some concerns”, primarily due to inadequate reporting of randomization and blinding. This substantially reduces the certainty of evidence, and our findings should be considered exploratory rather than confirmatory.

All trials were conducted in China, which limits the generalizability of our findings to other ethnic populations and healthcare systems. These findings are primarily applicable to the Chinese healthcare context and should not be directly extrapolated to other regions without further validation.

Future high-quality, large-scale trials are warranted to confirm the rankings observed in this NMA. Specifically, researchers should: (1) use standardized outcome measures (HAMD-17/24, response rate, and ADR checklists) with longer follow-up (≥6 months); (2) include active comparators to allow head-to-head comparisons between CPMs; (3) conduct trials outside China to enhance generalizability; (4) evaluate dose-response relationships and optimal treatment duration; (5) perform mechanistic studies (e.g., neuroimaging, inflammatory markers) to validate the hypothesized mechanisms; and (6) prioritise validation of YXQN+SSRIs in adequately powered trials.

## 5 Conclusion

In summary, this network meta-analysis suggests that adding Chinese patent medicines to SSRIs may enhance antidepressant efficacy and reduce adverse drug reactions. Among the seven CPMs evaluated, JYW+SSRIs had the highest SUCRA value for HAMD improvement, BJTGT+SSRIs showed a favorable SUCRA ranking for clinical response rate, and TM+SSRIs had the highest SUCRA value for safety, although none of the pairwise comparisons for ADR incidence reached statistical significance. YXQN+SSRIs also appears safe, but its evidence is extremely limited. These rankings reflect probabilities rather than proof of superiority and should be interpreted alongside effect estimates and confidence intervals. These hypothesis-generating findings offer exploratory insights that may inform treatment considerations, but should not be interpreted as supporting the superiority of any specific CPM–SSRI combination. However, the predominance of trials with some concerns in risk of bias limits the strength of conclusions, and as all included trials were conducted in China, these findings are primarily applicable to the Chinese healthcare context and should not be directly extrapolated to other regions without further validation. Due to methodological limitations and potential publication bias for the response rate outcome, large-scale, high-quality confirmatory trials are urgently needed.

## Data Availability

The original contributions presented in the study are included in the article/**Supplementary Material**, further inquiries can be directed to the corresponding author.
