# Correction: Comparative efficacy and safety of seven Chinese patent medicines combined with SSRIs for depressive disorder: a systematic review and network meta-analysis

**DOI:** 10.3389/fpsyt.2026.1972400

**Published:** 2026-09-04

**Authors:** Hongye Geng, Lixuan Li, Chengyuan Lu, Shuling Wang

**Affiliations:** School of Business Administration, Shenyang Pharmaceutical University, Shenyang, China

**Keywords:** adjunctive therapy, Chinese patent medicines, depressive disorder, network meta- analysis, selective serotonin reuptake inhibitors, systematic review

There was a mistake in **Table 1** as published. The original table did not clearly display the comparator group “SSRIs monotherapy” and omitted the corresponding mean differences (95% CI) for comparisons with certain interventions. To improve readability and facilitate correct interpretation of the network meta-analysis, the corrected **Table 1** now presents all interventions in a clear, side-by-side format against SSRIs monotherapy as the common reference. All numerical values remain unchanged from the original analysis; this correction does not affect the study's findings or conclusions. The corrected **Table 1** appears below.

**Table 1:** League table for HAMD improvement (MD, 95% CI).

**BJTGT+SSRIs**							
-1.42 [-5.11, 2.27]	**JYW+SSRIs**						
0.03 [-3.15, 3.21]	1.45 [-0.95, 3.85]	**SGJY+SSRIs**					
3.58 [0.58, 6.58]	4.99 [2.84, 7.15]	3.55 [2.49, 4.61]	**SSRIs**				
1.46 [-2.68, 5.60]	2.88 [-0.70, 6.45]	1.43 [-1.62, 4.47]	-2.12 [-4.97, 0.73]	**TM+SSRIs**			
1.12 [-2.83, 5.08]	2.54 [-0.82, 5.90]	1.10 [-1.69, 3.88]	-2.45 [-5.03, 0.13]	-0.33 [-4.18, 3.51]	**WL+SSRIs**		
0.92 [-2.60, 4.44]	2.34 [-0.49, 5.17]	0.89 [-1.23, 3.02]	-2.65 [-4.49, -0.82]	-0.54 [-3.93, 2.86]	-0.20 [-3.37, 2.96]	**XYW+SSRIs**	
1.68 [-4.76, 8.11]	3.09 [-2.99, 9.18]	1.65 [-4.14, 7.44]	-1.90 [-7.59, 3.79]	0.22 [-6.15, 6.58]	0.55 [-5.70, 6.80]	0.75 [-5.23, 6.73]	**YXQN+SSRIs**

Values in the upper-right triangle represent mean differences versus SSRIs monotherapy; values in the lower-left triangle represent pairwise comparisons between CPM+SSRI combinations. Negative values favor the column intervention.

Bold values indicate 95% CI does not include 0.

The original version of this article has been updated.

