# Supplementary material for: Comparative efficacy and safety of seven Chinese patent medicines combined with SSRIs for depressive disorder: a systematic review and network meta-analysis
Source: Front Psychiatry. 2026 Aug 14;17:1872413. doi: 10.3389/fpsyt.2026.1872413 (PMC13522133; doi:10.3389/fpsyt.2026.1872413)
Supplement: Supplementary file 1 [file Supplementaryfile1.docx]

Supplementary Material

**Figure and Table legends**

**Supplementary Table 1 Characteristics of included trials.**

| ***Table 1. Characteristics of included studies*** | | | | | | | | | |
| --- | --- | --- | --- | --- | --- | --- | --- | --- | --- |
| *All studies were parallel-arm RCTs conducted at a single center in China.* | | | | | | | | | |
|  |  |  |  |  |  |  |  |  |  |
| **Sertraline group (n=11)** | | | | | | | | | |
| **Study** | **Year** | **Diagnosis** | **Criteria** | **Intervention** | **Control** | **N** | **Male (%)** | **Duration** | **Blinding** |
| Ma Yuanye | 2009 | Depression + Anxiety | CCMD-3; HAMD ≥18 + HAMA ≥ 14 | Sertraline + Wuling | Sertraline | 132 | 43.9 | 6w | Unclear |
| Gao Yuedong | 2013 | depression | CCMD-3; HAMD ≥18 | Xiaoyao Wan + Sertraline | Sertraline | 76 | 56.6 | 8w | Unclear |
| Xu Dechun | 2016 | recurrent depression | Clinical criteria (recurrent) | Sertraline + Shugan Jieyu | Sertraline | 100 | 58.0 | 8w | Single-blind (patients) |
| Wang Fang | 2016 | depression | CCMD-3; HAMD-17 ≥17 | Sertraline + Wuling | Sertraline | 130 | 42.3 | 6w | Unclear |
| Bai Cunhai | 2018 | depression | Clinical criteria | Bajitian OSC + Sertraline | Sertraline | 98 | 41.8 | 6w | Unclear |
| Song Xinxin | 2019 | depression | Clinical criteria | Bajitian OSC + Sertraline | Sertraline | 80 | 48.8 | 10w | Open-label |
| Kang Yingmei | 2019 | depression | Clinical criteria | Jieyu Wan + Sertraline | Sertraline | 70 | 51.4 | 4w | Single-blind (assessors) |
| Chen Mingzhong | 2020 | Depression | ICD-10; TCM; HAMD-17 ≥17 | Xiaoyao Wan + Sertraline | Sertraline | 60 | 38.3 | 6w | Unclear |
| Jiang Wenbo | 2023 | depression | HAMD-17 score ≥ 17 | Shugan Jieyu + Sertraline | Sertraline | 60 | 56.7 | 8w | Single-blind (patients) |
| Zhang Dan | 2024 | depression | TCM: Liver-Qi stagnation. | Jieyu Wan + Sertraline | Sertraline | 60 | 43.3 | 8w | Unclear |
| Wu Jie | 2025 | depression | TCM: Heart-Kidney | Sertraline + Wuling | Sertraline | 79 | 43.0 | 8w | Unclear |
|  |  |  |  |  |  |  |  |  |  |
| **Fluoxetine group (n=10)** | | | | | | | | | |
| **Study** | **Year** | **Diagnosis** | **Criteria** | **Intervention** | **Control** | **N** | **Male (%)** | **Duration** | **Blinding** |
| Wang Xinfa | 2010 | MDD | DSM-IV; HAMD-17 >24 (inpt) | Jieyu Wan + Fluoxetine | Fluoxetine | 74 | 54.0 | 6w | Unclear |
| Zheng Lin | 2012 | depression | CCMD-3; HAMD-17 ≥17 | Shugan Jieyu + Fluoxetine | Fluoxetine | 68 | 38.2 | 12w | Unclear |
| Wang Lina | 2015 | depression | ICD-10; TCM; HAMD-17 ≥18 | Fluoxetine + Shugan Jieyu | Fluoxetine | 120 | 42.5 | 8w | Unclear |
| Zhang Meixia | 2016 | Depression + Insomnia | TCM: Liver-Qi; PSQI >7 | Fluoxetine + Shugan Jieyu | Fluoxetine | 58 | 46.6 | 6w | Unclear |
| Wang Ying | 2021 | depression | TCM: Liver-Spleen | Shugan Jieyu + Fluoxetine | Fluoxetine | 108 | 57.4 | 6w | Unclear |
| Xu Ting | 2021 | depression | CCMD-3 + TCM: Liver-Spleen | Xiaoyao Wan + Fluoxetine | Fluoxetine Hydrochloride | 86 | 53.5 | 6w | Unclear |
| Xie | 2023 | depression | NR | Tianmeng + Fluoxetine | Fluoxetine | 109 | 42.2 | 4w | Single-blind (patients) |
| Zhao Yuanyuan | 2023 | depression | DSM-5; HAMD-24 ≥21; TCM: Liver-Spleen | Fluoxetine + Shugan Jieyu | Fluoxetine | 64 | 48.3 | 6w | Unclear |
| Meng Fanfang | 2025 | depression | Clinical criteria | Jieyu Wan + Fluoxetine | Fluoxetine | 198 | 55.6 | 8w | Unclear |
| Li Yonghong | 2025 | depression | ICD-10; Duration >1 mo | Shugan Jieyu + Fluoxetine | Fluoxetine | 64 | 48.4 | 6w | Single-blind (patients) |
|  |  |  |  |  |  |  |  |  |  |
| **Escitalopram group (n=20)** | | | | | | | | | |
| **Study** | **Year** | **Diagnosis** | **Criteria** | **Intervention** | **Control** | **N** | **Male (%)** | **Duration** | **Blinding** |
| Liu Ji | 2013 | Severe depression | CCMD-3; HAMD ≥24 | Shugan Jieyu + Escitalopram | Escitalopram | 76 | 46.0 | 8w | Unclear |
| Shi Zejin | 2016 | depression | Clinical criteria | Shugan Jieyu + Escitalopram | Escitalopram | 60 | 46.7 | 6w | Unclear |
| Ke Feifei | 2016 | depression | CCMD-3; HAMD-17 17–28 + TCM: Liver-Spleen | Shugan Jieyu + Escitalopram Tablets | Escitalopram | 68 | 54.4 | 6w | Unclear |
| Li Qiangmin | 2016 | TRD | HAMD ≥17; Failed ≥2 ADs | Shugan Jieyu + Escitalopram | Escitalopram | 148 | 35.1 | 8w | Single-blind (patients) |
| Hao Jianhui | 2016 | depression | ICD-10; HAMD ≥17 | Tianmeng + Escitalopram Tablets | Escitalopram | 126 | 33.3 | 8w | Unclear |
| Diao Duanzhong | 2016 | depression | Clinical criteria | Escitalopram + Shugan Jieyu | Escitalopram | 134 | 49.2 | 8w | Unclear |
| Wu Hong | 2016 | Depressive episode | ICD-10: HAMD ≥18; HAMA ≥14 | Tianmeng + Escitalopram | Escitalopram | 60 | 51.7 | 8w | Unclear |
| Wang Jianchang | 2017 | depression | Clinical criteria | Bajitian OSC + Escitalopram Tablets | Escitalopram | 80 | 60.0 | 6w | Unclear |
| Wang Hongjie | 2018 | Depression + Anxiety | Clinical criteria (comorbid anxiety) | Shugan Jieyu + Escitalopram | Escitalopram | 90 | 43.3 | 6w | Open-label |
| Chen Lixian | 2020 | Mild-moderate depression | TCM: Liver-Spleen | Shugan Jieyu + Escitalopram Tablets | Escitalopram | 62 | 48.4 | 8w | Unclear |
| Huang Guozhe | 2020 | TRD | TRD criteria | Shugan Jieyu + Escitalopram | Escitalopram | 62 | 58.1 | 4w | Unclear |
| Jiang Xiying | 2021 | Depressive episode | ICD-10: Mild-moderate | Wuling + Escitalopram | Escitalopram | 40 | 47.5 | 6w | Unclear |
| Huang Changcan | 2022 | Depression + Anxiety | CCMD-3 | Shugan Jieyu + Escitalopram | Escitalopram | 84 | 46.4 | 8w | Unclear |
| Wei Zheng | 2023 | TRD | TRD (≥2 ADs failed) | Shugan Jieyu + Escitalopram | Escitalopram | 100 | 53.0 | 6w | Unclear |
| Wang Xin | 2024 | depression | Age 18–65, non-smoker | Shugan Jieyu + Escitalopram | Escitalopram | 65 | 30.8 | 8w | Unclear |
| Liu Feixue | 2024 | TRD | TCM: Liver-Spleen | Shugan Jieyu + Escitalopram | Escitalopram | 81 | 59.3 | 8w | Single-blind (patients) |
| Liu Fengchu | 2024 | Unipolar depression | Mild-mod; HAMD-17 17–24 | Shugan Jieyu + Escitalopram | Escitalopram | 120 | 60.0 | 8w | Single-blind (patients) |
| Zhang Xiabo | 2024 | TRD | DSM-5; HAMD ≥20 | Shugan Jieyu + Escitalopram Tablets | Escitalopram | 98 | 50.0 | 6w | Unclear |
| Su Feng | 2025 | depression | TCM: Liver-Spleen | Shugan Jieyu + Escitalopram | Escitalopram | 110 | 44.6 | 4w | Open-label |
| Li Xiangmin | 2025 | depression | TCM: Liver-Spleen | Jieyu Wan + Escitalopram | Escitalopram | 300 | 47.0 | 6w | Unclear |
|  |  |  |  |  |  |  |  |  |  |
| **Paroxetine group (n=16)** | | | | | | | | | |
| **Study** | **Year** | **Diagnosis** | **Criteria** | **Intervention** | **Control** | **N** | **Male (%)** | **Duration** | **Blinding** |
| Zhang Huadong | 2009 | depression | CCMD-3; HAMD-17 ≥18; TCM: Liver-Spleen | Paroxetine + Xiaoyao Wan | Paroxetine | 62 | 51.6 | 6w | Unclear |
| Li Li | 2012 | depression | CCMD-3; HAMD-17 ≥17 | Jieyu Wan + Paroxetine | Paroxetine | 60 | 35.0 | 8w | Unclear |
| Xiang Qin | 2012 | depression | CCMD-3 | Xiaoyao Wan + Paroxetine | Paroxetine | 76 | 67.1 | 12w | Unclear |
| Duan Xiancui | 2014 | Mild-moderate depression | Various subtypes | Wuling + Paroxetine | Paroxetine | 88 | 64.8 | 6w | Unclear |
| Zhang Yinghua | 2014 | Reactive depression | CCMD-3; HAMD-17 >24 | Xiaoyao Wan + Paroxetine | Paroxetine | 116 | 56.9 | 4w | Unclear |
| Huo Jun | 2015 | depression | Clinical criteria | Shugan Jieyu + Paroxetine | Paroxetine | 60 | 55.0 | 6w | Unclear |
| Cheng Yu | 2016 | depression | Clinical criteria (first-episode) | Shugan Jieyu + Paroxetine | Paroxetine | 60 | 58.3 | 8w | Unclear |
| Liu Dan | 2017 | Depression | ICD-10; First-episode; HAMD-17 >17 | Paroxetine + Xiaoyao Wan | Paroxetine | 60 | 48.3 | 6w | Unclear |
| Xu Daoxiang | 2017 | Depression + Anxiety | HAMD ≥20; HAMA ≥14 | Paroxetine + Xiaoyao Wan | Paroxetine | 160 | 58.1 | 8w | Single-blind (patients) |
| Guo Caie | 2017 | depression | CCMD-3; HAMD-17 ≥17 | Shugan Jieyu + Paroxetine | Paroxetine | 70 | 44.3 | 4w | Unclear |
| Zhang Wenjing | 2018 | Depression + Insomnia | CCMD-3; HAMD-24 ≥8 + PSQI ≥ 8 | Paroxetine + Yangxue Qingnao | Paroxetine | 60 | 45.0 | 8w | Unclear |
| Wang Rui | 2019 | depression | Clinical criteria | Shugan Jieyu + Paroxetine | Paroxetine | 94 | 56.4 | 8w | Unclear |
| Cao Chongyang | 2020 | depression | HAMD ≥18 | Bajitian OSC + Paroxetine | Paroxetine | 143 | 42.7 | 8w | Unclear |
| Chen Minmei | 2021 | depression | Clinical criteria | Shugan Jieyu + Paroxetine | Paroxetine | 60 | 56.7 | 8w | Unclear |
| Zhao Minqiang | 2021 | Depressive episode | Clinical criteria (episode) | Shugan Jieyu + Paroxetine | Paroxetine | 96 | 53.1 | 8w | Unclear |
| Shao Min | 2022 | depression | Clinical criteria | Bajitian Oligosaccharide + Paroxetine | Paroxetine | 116 | 33.6 | 8w | Unclear |
|  |  |  |  |  |  |  |  |  |  |
| **Citalopram group (n=4)** | | | | | | | | | |
| **Study** | **Year** | **Diagnosis** | **Criteria** | **Intervention** | **Control** | **N** | **Male (%)** | **Duration** | **Blinding** |
| Ma Cheng | 2007 | depression | CCMD-3; HAMD ≥17 | Xiaoyao Wan + Citalopram | Citalopram | 60 | 50.0 | 12w | Unclear |
| Yang Juan | 2013 | depression | CCMD-3; HAMD-17 ≥18 | Citalopram + Shugan Jieyu | Citalopram | 80 | 43.8 | 8w | Unclear |
| Gao Zhonglu | 2017 | depression | TCM: Liver-Spleen | Shugan Jieyu + Citalopram | Citalopram Hydrobromide | 132 | 51.5 | 12w | Unclear |
| Zhang Zhiqin | 2019 | TRD | TRD; HAMD ≥20 | Jieyu Wan + Citalopram | Citalopram | 87 | 55.2 | 8w | Unclear |
|  |  |  |  |  |  |  |  |  |  |
| **Fluvoxamine group (n=4)** | | | | | | | | | |
| **Study** | **Year** | **Diagnosis** | **Criteria** | **Intervention** | **Control** | **N** | **Male (%)** | **Duration** | **Blinding** |
| Zhou Donglin | 2012 | depression | CCMD-3; HAMD-17 ≥17 + TCM: Liver-Spleen | Fluvoxamine + Xiaoyao Wan | Fluvoxamine | 126 | 42.1 | 8w | Unclear |
| Wang Ping | 2015 | depression | TCM: Liver-Spleen | Shugan Jieyu + Fluvoxamine | Fluvoxamine | 72 | 48.6 | 8w | Unclear |
| Fan Changhe | 2016 | MDD | DSM-IV | Shugan Jieyu + Fluvoxamine | Fluvoxamine | 82 | 42.7 | 6w | Unclear |
| Qin Qiuhong | 2024 | depression | Clinical criteria | Wuling + Fluvoxamine | Fluvoxamine | 97 | 47.4 | 6w | Unclear |
| Abbreviations: RCT = randomized controlled trial; TCM = traditional Chinese medicine; TRD = treatment-resistant depression; MDD = major depressive disorder; HAMD = Hamilton Depression Rating Scale; HAMA = Hamilton Anxiety Rating Scale; PSQI = Pittsburgh Sleep Quality Index; NR = not reported; OSC = oligosaccharide capsule. | | | | | | | | | |
| Blinding: "Unclear" = unclear blinding; "Open-label" = open-label (no blinding); "Single-blind (patients)" = patients blinded; "Single-blind (assessors)" = outcome assessors blinded. | | | | | | | | | |

**Supplementary Tables 2A HAMD improvement**

| **Intervention** | **Studies** | **Heterogeneity** | **MD** | **95% CI** | **P** |
| --- | --- | --- | --- | --- | --- |
| BJTGT+SSRIs vs SSRIs | 3 | I²=0%, P=.889 | -5.01 | [-5.62, -4.39] | <0.001 |
| SGJY+SSRIs vs SSRIs | 29 | I²=96%, P=<0.001 | -3.47 | [-4.19, -2.74] | <0.001 |
| WL+SSRIs vs SSRIs | 5 | I²=47.1%, P=.109 | -2.3 | [-3.29, -1.31] | .003 |
| JYW+SSRIs vs SSRIs | 7 | I²=99.7%, P=<0.001 | -4.96 | [-8.51, -1.4] | .014 |
| XYW+SSRIs vs SSRIs | 9 | I²=83.4%, P=<0.001 | -2.63 | [-3.62, -1.64] | <0.001 |
| TM+SSRIs vs SSRIs | 3 | I²=66.7%, P=.050 | -1.2 | [-3.84, 1.44] | .189 |

**Supplementary Tables 2B Clinical response rate**

| Intervention | Studies | Heterogeneity | OR | CI_95 | P_value |
| --- | --- | --- | --- | --- | --- |
| SGJY+SSRIs vs SSRIs | 29 | I²=0%, P=0.991 | 3.06 | [2.58, 3.62] | <0.001 |
| XYW+SSRIs vs SSRIs | 10 | I²=0%, P=0.913 | 2.87 | [2.08, 3.95] | <0.001 |
| JYW+SSRIs vs SSRIs | 6 | I²=0%, P=0.629 | 2.98 | [1.78, 4.99] | 0.003 |
| BJTGT+SSRIs vs SSRIs | 5 | I²=18.5%, P=0.297 | 3.6 | [1.60, 8.07] | 0.012 |
| WL+SSRIs vs SSRIs | 5 | I²=0%, P=0.954 | 3.41 | [2.49, 4.66] | <0.001 |
| TM+SSRIs vs SSRIs | 3 | I²=0%, P=0.398 | 2.17 | [0.50, 9.52] | 0.152 |

**Supplementary Tables 2C ADR incidence**

| Intervention | Studies | Heterogeneity | OR | CI_95 | P_value |
| --- | --- | --- | --- | --- | --- |
| SGJY+SSRIs vs SSRIs | 24 | I²=16.6%, P=0.232 | 0.75 | [0.57, 0.98] | 0.034 |
| XYW+SSRIs vs SSRIs | 8 | I²=0%, P=0.535 | 0.43 | [0.30, 0.62] | 0.001 |
| JYW+SSRIs vs SSRIs | 6 | I²=65.8%, P=0.012 | 0.66 | [0.20, 2.16] | 0.404 |
| WL+SSRIs vs SSRIs | 5 | I²=21.9%, P=0.275 | 0.6 | [0.27, 1.32] | 0.147 |
| BJTGT+SSRIs vs SSRIs | 3 | I²=79.6%, P=0.007 | 0.37 | [0.01, 10.04] | 0.325 |
| TM+SSRIs vs SSRIs | 2 | I²=54.4%, P=0.139 | 0.55 | [0.00, 26770.86] | 0.614 |

**Supplementary Figures 1 Risk of bias summary (RoB 2.0) for all included trials.**

**Supplementary Figure 2 Risk of bias graph across all domains**
